# Research on the influence of employee psychological capital and knowledge sharing on breakthrough innovation performance

**DOI:** 10.3389/fpsyg.2022.1084090

**Published:** 2023-01-16

**Authors:** Yuhan Liu, Jianbin Chen, Xiaolei Han

**Affiliations:** ^1^Business College of Beijing Union University, Beijing, China; ^2^CRRC Industrial Academy Co., Ltd., Beijing, China

**Keywords:** employee psychological capital, knowledge sharing, breakthrough innovation performance, self-efficacy, emotional stability

## Abstract

Breakthrough innovation is the focus of the society in the era of knowledge economy. Employee innovation of the enterprises is the starting point of enterprise innovation behavior, and it is the result of the combination of complex psychological capital. Meanwhile, breakthrough innovation often comes from the result of knowledge sharing brought by teamwork. At present, existing studies mainly reveal the influence of knowledge and knowledge structure on the performance of radical innovation. However, the relationship between psychological capital, knowledge sharing and the breakthrough innovation performance needs to be systematically studied. Therefore, this study adopted a research approach, that is, statistical analyses were performed by using SPSS Version 18 and AMOS version 26 (Statistical analyses performed by using SPSS Version 18 and AMOS version 26).This study collected data on employees of 345 different new high-tech enterprises to explore the mechanism by which psychological capital and knowledge sharing affects the breakthrough innovation performance. The research results respond to a positive correlation between psychological capital and knowledge sharing affects the breakthrough innovation performance. Moreover, knowledge sharing has a mediating effect on the effect of psychological capital on breakthrough innovation performance, and the effect is weakened. which is of great theoretical significance for exploring the relationship between psychological capital and knowledge sharing affects the breakthrough innovation performance.

## 1. Introduction

In an era of knowledge-based economy, innovation is the focus topic in recent years, especially the breakthrough innovation, the research of which has increased greatly in recent years. Tu Youyou is the first top scientific researcher in China to win the Nobel Prize in Physiology or Medicine. She has made breakthrough innovation and outstanding contributions to the development of the world’s medical and health undertakings. Breakthrough innovation exists in line with incremental innovation and is usually accompanied by technological leapfrogging and substitution. Due to the progress of science and technology, and the short cycle of R&D in terms of technology and products, the competitive advantages brought by breakthrough innovation for enterprises have feature more prominently. Ke and Kunji (2013) successfully link breakthrough innovation with core competitiveness from the perspective of resources. Charles and Rothaermel (2003) focus on the management model and operation mechanism of breakthrough innovation enterprises. A large number of studies have shown that improving the performance of breakthrough innovation is the only way for countries, organizations and groups to win competitive advantages. However, there is little empirical analysis on the performance of breakthrough innovation. The existing research mainly focuses on alliance, network, resources and other fronts. For example, the research of Taishan and Yulin (2016) find that close alliance network relationship is an important means to promote breakthrough innovation. Research and development cooperation can significantly enhance the breakthrough innovation of enterprises, and knowledge pool is positively affecting the breakthrough innovation performance of alliance enterprises. Jun et al. (2014) construct a model from the perspective of “network capability-organization tacit knowledge acquisition-breakthrough innovation performance.” The research found that network capability has a significant positive impact on breakthrough innovation performance, and organization tacit knowledge acquisition mediates the relationship between the two. Jian et al. (2010) integrate the resource-based theory and the resource allocation theory to study the driving mechanism of key resources on the breakthrough innovation performance of international companies in China.

Only by constantly enhancing the enterprise’s breakthrough innovation vitality can the global enterprises get better development and have a positive impact on the global development. Breakthrough innovation can be regarded as a process of “knowledge acquisition-knowledge flow-knowledge creation.” The main source of breakthrough innovation in an enterprise is employees. To improve the performance of breakthrough innovation of employees, one cannot only consider one factor, but also need to be specific to each stage of breakthrough innovation. Psychological capital is considered to be the key determinant of the competitive advantage of the future organization and a truly valuable asset of the enterprise. The main purpose of knowledge sharing is to increase the mobility of creativity. At present, the research on knowledge and breakthrough innovation mainly focuses on knowledge foundation, knowledge conflict, knowledge strategy and other aspects. For example, Beiling et al. (2012) study the impact of intellectual capital on the performance of enterprise breakthrough innovation from the perspective of knowledge; Zhiming (2016) conducts an empirical study on the relationship among corporate knowledge base, open innovation and breakthrough innovation performance; Huibin and Daming (2014) conduct an empirical study on the relationship among team knowledge conflict, organizational learning and breakthrough innovation performance; Xiaofen and Qiang (2017) conduct an empirical analysis on the impact of external knowledge sourcing strategy, actual and potential absorptive capacity on the performance of breakthrough innovation. To a certain extent, these studies reveal the impact of knowledge and knowledge structure on the performance of breakthrough innovation. However, the relationship among psychological capital, knowledge sharing and the performance of breakthrough innovation still needs to be studied systematically.

In summary, combining the realistic background and theoretical background mentioned above, we can find that despite the strong link between psychological capital, knowledge sharing and breakthrough innovation performance, existing research has not paid enough attention on this domain. Base on the existing research, this study conducts a theoretical discussion and empirical analysis on the impact of psychological capital and knowledge sharing on the breakthrough innovation performance. Moreover, in order to enrich and develop the research on the intermediary role of knowledge sharing between psychological capital and breakthrough innovation performance, knowledge sharing was introduced as a mediator variable to study the intermediary role of knowledge sharing between psychological capital and breakthrough innovation performance. This study mainly focuses on the following two questions: RQ1: As a kind of highly subversive and uncertain innovation, is its performance affected by psychological capital as well as innovation performance? Namely, the relationship between psychological capital and breakthrough innovation performance. RQ2: Does knowledge sharing play an intermediary role in the impact of psychological capital on breakthrough innovation performance, and what is the magnitude and direction of the intermediary effect? I.e. the exploration of the intermediary role of knowledge sharing.

## 2. Theoretical background and hypotheses development

### 2.1. Research on the breakthrough innovation performance

According to the research on the performance of breakthrough innovation, Abemathy and Utterback (1978) first proposed the concept of breakthrough innovation. Different from continuous innovation, breakthrough innovation takes the potential market as a breakthrough. Its technological development path is not to improve on the original technological track but to find a new way. It breaks the competitive basis of the original technology and thus becomes an important way for enterprises to realize technological leapfrogging and obtain continuous competitive advantages. Breakthrough innovation is the result of a systematic study, which focuses on the reorganization and creation of knowledge (McDermott and O'Connor, 2002) and is characterized by discontinuity and nonlinearity (Linton, 2009).

From the research on the knowledge management, Huibin and Daming (2014) study the relationship among team knowledge conflict, organizational learning and breakthrough innovation performance, and found that team conflict can have an effect on breakthrough innovation performance, while organizational learning plays an intermediary role in the relationship between them. Similarly, from the perspective of knowledge, Ling et al. (2012) find that human, social and relational capital in intellectual capital can positively affect the breakthrough innovation performance of the company. In conclusion, it can be seen that team knowledge learning has an impact on the company’s breakthrough innovation performance.

From the measurement research of breakthrough innovation performance, Daming and Beiling (2014) measure breakthrough innovation performance from two aspects: process and product. Yim and Tse (2005) think that breakthrough needs have the functions of brand-new product performance and exploring potential customer demand and increasing customer value according to the characteristics of breakthrough innovation. Therefore, the performance of breakthrough innovation is measured by two measurement items: product quality and customer value realization. Similarly, Ke and Kunji (2013) point out that the measurement of breakthrough innovation performance should not simply focus on the external observable final results, where the most direct embodiment of the final results is the new products, technologies or services produced. This measurement method ignores the new way of thinking, pioneering innovative ideas and the new discovery and use of scarce resources. Furthermore, the research of Junjie et al. (2017) and Yang et al. (2014) measure the items from three aspects: the development of new technology, products and services, and the impact on existing knowledge and experience. In conclusion, there are mainly three aspacts about the measurement research of breakthrough innovation performance, such as product, process and knowledge.

### 2.2. Research on the psychological capital

Psychological capital refers to an individual’s psychological state in the process of growth and development. It is a core psychological element beyond human capital and social capital and a psychological resource to promote personal growth and performance improvement (Xudong, 2017). It is a combination of individuals’ beliefs about themselves, social relationships, career development, morality, life goals, and life (Goldsmith et al., 1997). Similarly, Seligman (2002) believes that psychology focuses on the individual’s advantages, health, vitality and other aspects. Furthermore, Luthans et al. (2004) believes that psychological capital is a kind of psychological ability, which includes confidence, hope, optimism and resilience. In conclusion, psychological capital is a core psychological element, a psychological resource, and psychological ability.

On the measurement of psychological capital, Goldsmith et al., 1997 divides psychological capital into two aspects: control and self-esteem. Furthermore, Luthans et al. (2008) classifies psychological capital into four levels: self-confidence/self-efficacy (having the confidence to make the necessary efforts to achieve success in the face of challenging work), hope (persevering in the pursuit of goals and adjusting the way to achieve them when necessary), optimism (maintaining a positive attitude towards the present and the future), and resilience (being firm, energetic and successful when troubled by difficulties and adversities).Similarly, Letcher (2003) divides psychological capital into five dimensions: extroversion, emotional stability, agreeableness, openness and responsibility. Similarly, Cole (2006) believes that psychological capital is self-esteem, self-efficacy, control points and emotional stability. Therefore, this study defines psychological capital as a state of mind in which an individual has a relatively stable emotional state and has the ability to recognize himself and believe in the knowledge and skills one has. It is divided into self-efficacy and emotional stability. Among this research conclusion, Goldsmith et al. (1997) divided psychological capital into two aspects: control and self-esteem. Subsequently, some scholars divided psychological capital into three, four or five dimensions. Among them, the most mainstream is the theory proposed by Luthans (2005), who divides it into self-efficacy, hope, resilience and optimism. However, some scholars believe that there are dimensionality and similarity in psychological capital. Hope, resilience and optimism belong to three items of emotional stability. Therefore, our study divides psychological capital into self-efficacy and emotional stability.

### 2.3. Research on the knowledge sharing

From the perspective of the concept of knowledge sharing, Bartol and Srivastava (2002) believe that knowledge sharing is a process of information transmission, in which individuals in an organization put forward their own views and suggestions to others, or share their experience and skills to others. Knowledge sharing is the transfer or flow of knowledge between different individuals and organizations (Lee, 2001). Knowledge sharing aims at knowledge appreciation. Members of an organization share the information obtained from various channels.

Judging from the measurement of knowledge sharing, different scholars divide knowledge sharing differently. One-dimensional partitioning, such as Chowdhury’s (2005) complex knowledge sharing, emphasizes the process of knowledge sharing. Two-dimensional division, for example, Zarraga and Bonache (2003) divide knowledge sharing into knowledge transfer and knowledge creation, which includes both the flow of knowledge and the generation of new knowledge. Furthermore, Hooff and Bidder (2004) put forward what is two-dimensional division, including knowledge contribution (giving knowledge to others) and knowledge collection (searching, learning, integrating knowledge), implicit and explicit knowledge sharing, and internal and external knowledge sharing of the organization; efforts and frequency of knowledge sharing (Bock et al., 2005; Mooradian et al., 2006; King, 2008). The research on the influencing factors of knowledge contribution is mainly divided into individual factors (Wansong et al., 2014) and organizational factors (Pee et al., 2010).

Therefore, knowledge sharing is a complicated process, the first is to collect and accumulate knowledge and have a certain knowledge reserve. The second is to share and exchange one’s own views, skills and ideas with others. The purpose of knowledge sharing is to increase the flow of ideas. At the same time, individual factors (personal image, personal motivation, etc.) and organizational factors (organizational culture, interpersonal relationships between organizations, etc.) will affect knowledge sharing behavior.

### 2.4. Analysis of the influence of psychological capital, knowledge sharing on breakthrough innovation performance

#### 2.4.1. Employee’s psychological capital and knowledge sharing

Cabrera et al. (2006) find that self-efficacy is a variable that can promote knowledge exchange. People with self-confidence are more willing to speak out their own opinions. Avey et al. (2011) believe that there may be some connection between psychological capital and knowledge sharing. Threshold (2011) finds that psychological capital can positively predict knowledge sharing behavior. Abella and Zapata (2011) find that employees who are full of hope, optimistic about life and strong willpower are more likely to share knowledge. On the other hand, Bansemir et al. (2012) believe that employees’ individual will has a key impact on knowledge sharing behavior. Combs et al. (2017) believe that employees’ psychological capital can influence their willingness to share knowledge, and employees with higher psychological capital are more willing to share knowledge. Qian and Minggui (2014) find a positive correlation between positive psychological capital and knowledge sharing. There are barriers to knowledge sharing, and employees with a positive attitude can put themselves into work in a positive and full state and break the barriers. Goldsmith et al. (1997) divided psychological capital into two aspects: control and self-esteem. Subsequently, some scholars divided psychological capital into three, four or five dimensions. Among them, the most mainstream is the theory proposed by Luthans (2005), who divides it into self-efficacy, hope, resilience and optimism. However, some scholars believe that there are dimensionality and similarity in psychological capital. Hope, resilience and optimism belong to three items of emotional stability. Therefore, our study divides psychological capital into self-efficacy and emotional stability. Zhengde (2018) finds that employees with high self-efficacy can continuously strengthen their study, actively seek new shortcuts, and prefer to share new knowledge. Emotional stability can stabilize the innovative spirit of the employees, and make the innovative spirit of the employees play the most effective role in the process of improving the breakthrough innovation performance. From the perspective of the relationship between emotional stability and leadership, Hui et al. (2019) think that emotional stability can ensure the knowledge creation and knowledge sharing of employees. The degree of knowledge sharing increases with the increase of emotional stability (Hui et al., 2019). Therefore, the following assumptions are made:

*H1:* The self-efficacy of employees’ psychological capital has a significant positive impact on knowledge sharing.

*H2:* The emotional stability of employees’ psychological capital has a significant positive impact on knowledge sharing.

#### 2.4.2. Employee’s psychological capital and breakthrough innovation performance

The performance of enterprise’s breakthrough technological innovation is closely related to the employee’s psychological capital level. Employee with high psychological capital level can actively accept knowledge, define the technological innovation goal, and put the expectation of the future into action, which reflects higher motivation (Qingsong et al., 2018). Larson et al. (2008) believe that employees who have hope are better able to achieve their goals because they are able to develop more practical action plans, thus it seems that employees who have hope are better able to achieve breakthrough innovation. Carr (2008) finds that employee psychological capital can better explain the performance of breakthrough innovation than human capital and social capital, and psychological capital is a variable that can explain the performance of breakthrough innovation. Qingsong and Daming (2010) find that a healthy, positive and sunny attitude of employees can help to generate breakthrough technological innovation performance for enterprises. In addition, breakthrough innovation is risky, and people with optimism and strong willpower are more likely to achieve innovation. Carr (2008) finds that optimistic employees are more receptive to new ideas and showed more breakthrough creativity. Luthans et al. (2008) also points out that employees with strong will are better able to meet the challenges at work and achieve breakthrough success. Yuan and Jun (2016) find that psychological capital has a positive effect on employees’ initiative of breakthrough innovation. Linying (2017) finds that the six dimensions of team psychological capital, namely, self-confidence, hope, optimism, resilience, cooperation and responsibility, can all promote breakthrough innovation performance. Leadership trait theory holds that in an organization, leaders’ attention to subordinates can enhance employees’ sense of self-efficacy and thus enhance employees’ innovative behavior (Qing et al., 2022). People’s creativity stems from self-efficacy. Emotional stability has a significant impact on employees’ breakthrough innovation performance. Employees show a relatively stable emotional response after receiving external stimuli, and can recover to normal emotional level more quickly to improve their breakthrough innovation performance (Xiaoxia and Rui, 2012). In the spirit of innovation, the more stable the employee’s mood and the more accurate the judgment on the market, the more significant the breakthrough innovation performance improvement will be. Therefore, the following assumptions are made:

*H3:* The self-efficacy of employees’ psychological capital has a significant positive impact on breakthrough innovation performance.

*H4:* The emotional stability of employees’ psychological capital has a significant positive impact on breakthrough innovation performance.

#### 2.4.3. Knowledge sharing and breakthrough innovation performance

Liebowitz (2002) believes that knowledge sharing behavior can promote the improvement of breakthrough innovation ability, and improve the breakthrough innovation ability and performance of organizations. Syed-Ikhsan (2004) et al. believe that knowledge sharing is the key factor to improve the enterprise’s ability, that is, knowledge sharing helps to improve the ability of breakthrough innovation. CameloOrdaz et al. (2011) indicates a positive relationship between knowledge sharing and innovation performance. Qianjun (2013) find that knowledge sharing behavior is the key condition to achieve breakthrough innovation performance. Zifen and Yue (2013) believe that knowledge sharing has a direct positive impact on employees’ breakthrough innovation behavior. Han and Chen (2016) find that the exploratory knowledge sharing behavior significantly promoted the performance of breakthrough innovation, and the transmission of knowledge could improve the performance of breakthrough innovation. Therefore, the following assumptions are made:

*H5:* Knowledge sharing has a significant positive impact on employees’ breakthrough innovation performance.

#### 2.4.4. Employee’s psychological capital, knowledge sharing and breakthrough innovation performance

**Figure 1:**
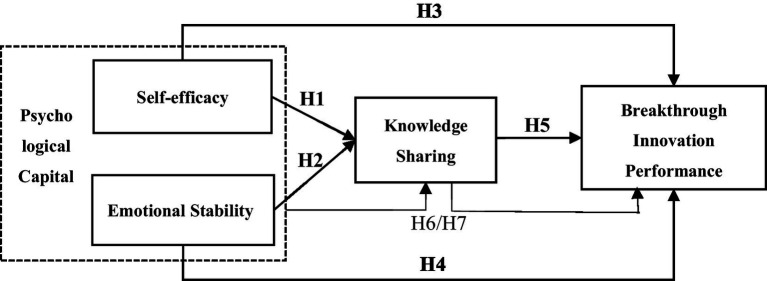
Research framework.

Fenglian (2014) finds that employees with a positive attitude are more likely to share and exchange knowledge with others, and ultimately promote the breakthrough innovation of employees. Wei (2015) believes that attaching importance to employees’ psychological capital and cultivating employees’ ability to acquire knowledge can bring better breakthrough innovation performance. In addition, knowledge sharing often appears as an intermediate variable in relevant research (Figure 1). For example, knowledge sharing is mediated between organizational trust and performance of new products (Vanguard et al., 2010). Between internal social capital and employee’s breakthrough innovation behavior (Zifen and Yue, 2013). The relationship between team psychological security and employees’ breakthrough innovation behavior (Keyan, 2015). Between positive emotions and team creativity, emotions will affect team members’ tacit knowledge sharing behavior, thus affecting breakthrough creativity (Chaoying et al., 2011). Therefore, the following assumptions are made:

*H6:* Knowledge sharing plays an intermediary role in the relationship between self-efficacy of psychological capital and breakthrough innovation performance.

## 3. Materials and methods

### 3.1. Sample and data

The questionnaire for this study was distributed from January 2022 to April 2022, and lasted for 4 months. This item defines the measurement of breakthrough innovation performance as the performance generated when middle and senior managers of high-tech enterprises and grassroots employees committed to R&D participate in enterprise innovation. The respondents of the questionnaire are high-tech enterprises, including the middle and senior managers of the enterprises and the grass-roots employees who are committed to R&D. The focus on high-tech enterprises is determined by the research issues in this study. The purpose of this research is to study the relationship among psychological capital, knowledge sharing and breakthrough innovation performance, which requires the selected samples to have breakthrough innovation behavior and truly reflect breakthrough innovation performance. High-tech enterprises, on the other hand, are based on major technological breakthroughs and major development needs, thus ensuring the emergence of breakthrough innovations. Of course, not all enterprises have achieved breakthrough innovation success, and there are also failed enterprises. The breakthrough innovation of these enterprises can also reflect the research model of this paper. As the decision-maker of the enterprise, the middle and senior managers of the enterprise have a comprehensive grasp and control of the enterprise, can better understand the development status and the environment of the enterprise, and fill in the questionnaire with higher authenticity and representativeness. The grass-roots staff cannot know enough information about the firm level, it has certain limitations. However, because the grass-roots staff in deep production or research and development also have a profound understanding of the firm’s breakthrough innovation, it still has a certain reference. Enterprises pay great attention to the breakthrough innovation performance, and there are many shortcomings in this aspect, so we could tell the managers or R&D employees of enterprises when handing out questionnaires that we would provide suggestions for the improvement of breakthrough innovation performance based on the conclusions of this study, so as to encourage enterprises to be willing to participate in this study and ensure the quality of questionnaire filling. This questionnaire is mainly distributed to high-tech enterprises in the Beijing-Tianjin-Hebei region. In principle, each enterprise is required to fill in only one questionnaire as a sample. According to the availability and operability of the questionnaire, the electronic questionnaire is the main one, and the paper questionnaire is the supplementary one. The returned questionnaire was selected manually to further ensure its authenticity and representativeness. Among them, the paper-based questionnaire is mainly to eliminate the questionnaires with missing values, abnormal values and one option selected throughout or intermittently. The electronic version of the questionnaire mainly eliminates outliers, selects an option throughout or intermittently, and answers the questionnaire within 30 s. After the above-mentioned data collection and simple processing, the result is that 400 questionnaires are issued and 367 questionnaires are recovered, among which 345 were valid questionnaires, with a total effective recovery rate of 86.25%.

By calculating the descriptive statistical analysis of the questionnaires, this study draws the following analysis based on the valid questionnaires returned: There are 235 men, accounting for about 60.1%, which is mainly because the survey object of this sample is mainly managers, and managers in high-tech enterprises, especially middle and senior managers, are mostly men. The positions of the employee are distributed throughout the ordinary employee, grass-roots management, middle-level management and senior management, accounting for 12%, 25.3%, 36.1%, and 26.6% respectively, among which the middle-level and senior management are the leading ones, ensuring the authenticity of the sample and reflecting the real situation of the enterprise to a greater extent. Private enterprises and joint ventures are the main forms of ownership, and some state-owned enterprises and wholly foreign-owned enterprises are in line with the current development layout of high-tech industries. The establishment period is divided into stages with intervals of 10 years, and each stage is evenly distributed. The number of employees is divided into stages with an interval of 100, among which 200–300 are the majority, and the overall distribution is normal. The industry types are mainly high-tech enterprises, and each industry is evenly distributed. In general, the sample data of this study has a wide research scope and reasonable structure, which is highly associate to the research design requirements. Table 1 shows the descriptive statistical analysis results of samples’ basic characteristics.

**Table 1:** Descriptive statistical analysis of basic characteristics of samples.

Demographic variables		Frequency	Percentage%	Cumulative percentages
Gender	Female	110	31.9	31.9
	Male	235	68.1	100.0
Marital status	Married	215	62.3	62.3
	Unmarried	130	37.7	100.0
Culture level	Below Bachelor	97	28.1	28.1
	Bachelor	192	55.7	83.8
	Master	52	15.1	98.8
	Doctor	4	1.2	100.0
Working years	Below 1 year	80	23.2	23.2
	2–4 years	129	37.4	60.4
	5–7 years	29	5.8	66.2
	8–10 years	20	23.2	89.3
	Above 10 years	87	25.2	100.0
Age	18–25	153	44.4	44.4
	26–30	95	27.5	71.9
	31–40	42	12.2	84.1
	41–50	33	9.6	93.7
	51–60	22	6.3	100.0
Position	Ordinary staff	47	12.0	12.0
	junior managers	99	25.3	37.3
	Middle Manager	141	36.1	73.4
	Senior Managers	104	26.6	100.0
Forms of ownership	State-owned enterprise	83	21.2	21.2
	Private enterprise	124	31.7	52.9
	Ioint venture enterprise	117	29.9	82.9
	Foreign-owned enterprise	67	17.1	100.0
Establishment time	0–10 years	60	15.3	15.3
	11–20 years	89	22.8	38.1
	21–30 years	81	20.7	58.8
	31–40 years	91	23.3	82.1
	Above 40 years	70	17.9	100.0
Number of employees	Below 100	56	14.3	14.3
	101–200	95	24.3	38.6
	201–300	105	26.9	65.5
	301–400	73	18.7	84.1
	Above 400	62	15.9	100.0
Industry type	Information technology industry	76	19.4	19.4
	Bioindustry	136	34.8	54.2
	New material industry	77	19.7	73.9
	New energy industry	56	14.3	88.2
	Other	46	11.8	100.0

### 3.2. Measures

In this study, the five-point Likert scale was used to measure the multi-index variables involved in the questionnaire. More precisely, the respondents of the questionnaire make subjective scores according to the items measured in the questionnaire. The higher the score, the greater the tendency to agree. A score of one means strongly disagree while a score of five means strongly agree. The overall Cronbach’s value of the scale is 0.932.

#### 3.2.1. Dependent variable

The Breakthrough Innovation Performance (BIP) is measured by the Innovation Performance Scale compiled by Janssen (2000), Junjie et al. (2017), and Yang et al. (2014), which includes three dimensions, i.e., the generation, promotion and realization of innovation ideas, with a total of four items. The Likert’s 5-level scale is adopted in this study to measure and conducts the analysis with several adjustment according to the research situation. A Total of four items were used in the measurement of breakthrough innovation performance. Typical topics are “your company often introduces new ideas in product development,” “your company often creates products with new performance,” “your company develops and introduces new production technologies in the industry,” “your company creates new technological processes.” The scale Cronbach’s value is 0.951.

#### 3.2.2. Independent variable

Psychological Capital (PC) mainly includes two dimensions, which are self-efficacy and emotional stability. The specific measurement methods are as follows:

1. Self-efficacy (SE) lies in the degree of confidence in one’s own abilities and future expectations, while those who have received higher education and have a good educational background can endow themselves with the courage to face problems and the confidence to solve them when they are in trouble. Self-efficacy was measured by the self-efficacy Scale compiled by Jianguo (2006), Zhui and Qian (2016), and Qing et al. (2022), with six items in total. The Likert’s 5-level scale is adopted in this study to measure and conducts the analysis with several adjustment according to the research situation. A Total of six items were used in the measurement of self-efficiency. Typical topics are “I can confidently express my opinions on company planning,” “I can get out of work difficulties,” “I can confidently analyze and solve problems,” “I can do my best to achieve my work goals,” “I can keep energetic in my work” and “all problems have solutions.” The scale Cronbach’s value is 0.876.
2. Emotional stability (ES) will affect employees’ job satisfaction, which will lead them to identify with and trust leaders and organizations, and pay attention to their own responsibilities. The emotional stability is measured by the emotional stability scale prepared by Hui et al. (2019) and Xiaoxia and Rui (2012), with 5 items in total. The Likert’s 5-level scale is adopted in this study to measure and conducts the analysis with several adjustment according to the research situation. Typical topics are “I can face the work pressure calmly,” “I can stand firm when facing the work difficulty,” “I can deal with many things at the same time,” “I am optimistic about the uncertain things,” “I am optimistic about the future,” “I think there are two sides to everything and there is no need to be pessimistic.” The scale Cronbach’s value is 0.882.

#### 3.2.3. Intermediate variable

The measurement of Knowledge Sharing (KS) is based on a scale of 10 items compiled by Ardichvil et al. (2003), Hooff and Bidder (2004) and Weggeman (2004). The Likert’s 5-level scale is adopted in this study to measure and conducts the analysis with several adjustment according to the research situation. Typical topics are “when I learn something new, my colleagues in the department can also learn it,” “I share the information I have with my colleagues in the department,” “I share my skills with my colleagues in the department,” “When I learn something new, my colleagues outside the department can also learn it,” “I share the information I have with my colleagues outside the department,” “I share my skills with my colleagues outside the department,” “when I ask my colleagues inside the department, they will tell me what they know,” “when I ask my colleagues inside the department, they will tell me about their skills,” “when I ask my colleagues outside the department, they will tell me what they know,” “when I ask my colleagues outside the department, they will tell me about their skills.” The scale Cronbach’s value is 0.919.

### 3.3. Data quality test

#### 3.3.1. Confirmatory factor analysis

In order to verify the convergent validity of the model and the discriminant validity of key variables, this study adopted AMOS 26.0 software for confirmatory factor analysis. As listed in Table 2, confirmatory factor analysis was applied to analyze the factor structure of self-efficacy, emotional stability, knowledge sharing, breakthrough innovation performance, indicates that the 4-factor model has a good fit that *χ*^2^*/df* = 2.617, *CFI* = 0.858, *RMSEA* = 0.066, *AGFI* = 0.853, *GFI* = 0.840, *NFI* = 0.973.

**Table 2:** Results of the confirmatory factor analysis.

Models	x^2^/df	CFI	RMSEA	AGFI	GFI	NFI
(Benchmark model) 4-Factor	2.617	0.858	0.066	0.853	0.840	0.973
3-Factor model	1.73	0.931	0.059	0.890	0.967	0.933
2-Factor model	4.375	0.837	1.022	0.842	0.889	0.886
1-Factor model	1.922	0.899	0.059	0.891	0.923	0.916

*N* = 345.

4-factor model: Self-efficacy, Emotional Stability, Knowledge Sharing, Breakthrough Innovation Performance.

3-factor model: combines Self-efficacy and Emotional Stability into one factor based on the benchmark model.

2-factor model: combines Self-efficacy, Emotional Stability and Breakthrough Innovation Performance based on the benchmark model.

Single-factor model: combines Self-efficacy, Emotional Stability, Breakthrough Innovation Performance and Knowledge Sharing into one factor based on the benchmark model.

#### 3.3.2. Reliability and validity tests

The reliability emphasizes the reliability, credibility and stability of the measurement results. The collected data are processed by SPSS18.0, and the Cronbach’s alpha is used to test the reliability of the above scales. According to the judgment principle of internal consistency coefficient, that is, when the coefficient of the whole scale detected by the data is above 0.5, the set of scales can be used, and when the coefficient is above 0.5 and below 0.7, the reliability of the set of scales is ideal. The coefficient above 0.8 is ideal. According to the reliability analysis results in Table 3, Cronbach’s alpha of the two dimensions of psychological capital, namely self-efficacy and emotional stability, is 0.897 and 0.905 respectively, among which Cronbach’s alpha of emotional stability is >0.9, indicating that its reliability is very good. The Cronbach’s alpha of self-efficacy is above 0.8, indicating its reliability is very good. The CR of each dimension is >0.7, with good internal consistency. The Cronbach’s alpha of knowledge sharing is 0.912, which is >0.9, indicating that its reliability is very good, and CR is >0.7, which has good internal consistency. The Cronbach’s alpha of the breakthrough innovation performance is 0.905, which is >0.9, indicating that its reliability is very good, and the CR is >0.7, which has good internal consistency.

**Table 3:** Reliability analysis table of the questionnaire.

Variable	Dimension	Item	CITC	Cronbach’s alpha if item deleted	Cronbach’s alpha based on standardized items	Composite reliability
Psychological capital	Self-efficacy	Q1-1	0.669	0.883	0.897	0.898
		Q1-2	0.789	0.865		
		Q1-3	0.684	0.882		
		Q1-4	0.742	0.872		
		Q1-5	0.729	0.873		
		Q1-6	0.692	0.879		
	Emotional stability	Q2-1	0.695	0.857	0.905	0.915
		Q2-2	0.672	0.860		
		Q2-3	0.634	0.869		
		Q2-4	0.647	0.864		
		Q2-5	0.795	0.839		
Knowledge sharing		Q3-1	0.682	0.520	0.912	0.866
		Q3-2	0.746	0.713		
		Q3-3	0.769	0.578		
		Q3-4	0.715	0.739		
		Q3-5	0.794	0.682		
		Q3-6	0.807	0.697		
		Q3-7	0.802	0.731		
		Q3-8	0.821	0.786		
		Q3-9	0.825	0.775		
		Q3-10	0.864	0.824		
Breakthrough innovation performance		Q4-1	0.797	0.818	0.905	0.906
		Q4-2	0.772	0.852		
		Q4-3	0.769	0.863		
		Q4-4	0.814	0.851		

As the scales selected in this study all come from the mature research results of scholars in relevant fields, and experts and teachers in this field are invited to check the contents of the measurement items, pass the pre-test and modify the problems, the questionnaire has good content validity. Secondly, KMO and Battlett tests are performed on the scale. First of all, the psychological capital scale is tested for the sphericity of KMO and Battlett, and KMO = 0.965 (>0.9), indicating that this variable is very suitable for factor analysis. The observed value of the Battlett sphericity test is 5733.221, and the *p*-value = 0.0007, which is less than the given significant level of 0.01, indicating that there is correlation between the variables. Secondly, the knowledge sharing scale is tested by KMO and Barflett sphericity, and KMO = 0.886 (close to 0.9), indicating that this variable is very suitable for factor analysis. The observed value of the Battlett sphericity test is 3005.682, and the *p*-value = 0.0003, which is less than the given significant level of 0.01, indicating that there is correlation between the variables. Thirdly, KMO and Bartlett’s sphericity test are carried out on the Breakthrough Innovation Performance Scale, and KMO = 0.982 (>0.9), indicating that this variable is suitable for factor analysis. The observed value of Barmlets sphericity test is 2780.953, and the *p*-value = 0.0002, which is less than the given significant level of 0.01, indicating that there is correlation between the variables.

#### 3.3.3. Descriptive statistics and correlation analysis of the research variable

The purpose of this study is to explore the relationship between various variables. Before using the structural equation model for hypothesis testing, the correlation between various variables is tested. In this paper, Pearson correlation analysis method is used to test the relationship between various variables (Table 4). Table 4 gives Pearson correlation coefficient between each variable. The results shows that the two dimensions of psychological capital (self-efficacy and emotional stability) are significantly positively correlated with knowledge sharing (*r* = 0.755, *p* < 0.01; *r* = 0.672, *p* < 0.01). The two dimensions of psychological capital (self-efficacy and emotional stability) are significantly positively correlated with breakthrough innovation performance (*r* = 0.783, *p* < 0.01; *r* = 0.739, *p* < 0.01). In addition, knowledge sharing is significantly positively correlated with breakthrough innovation performance (*r* = 0.623; *p* < 0.01). The correlation coefficient of each variable is <0.8, and there is no serious collinearity problem between each variable. All dimensions of the four variables are significantly correlated.

**Table 4:** Variable mean, standard deviation, correlation coefficient and mean extraction variance (*N* = 345).

	SE	ES	KS	BIP
SE	(0.65)			
ES	0.755^**^	(0.72)		
KS	0.517^**^	0.672^*^	(0.78)	
BIP	0.783^**^	0.739^**^	0.623^*^	(0.61)
Mean	3.35	4.21	3.29	2.01
SD	0.631	0.451	0.373	0.312

*p < 0.05; **p < 0.01.

The value in brackets is the average variance extracted (AVE) of each factor. The corresponding dimension factor standard accords with the average value of the sum of squares.

## 4. Structural equation model and regression analysis

### 4.1. Data calculation and processing

**Figure 2:**
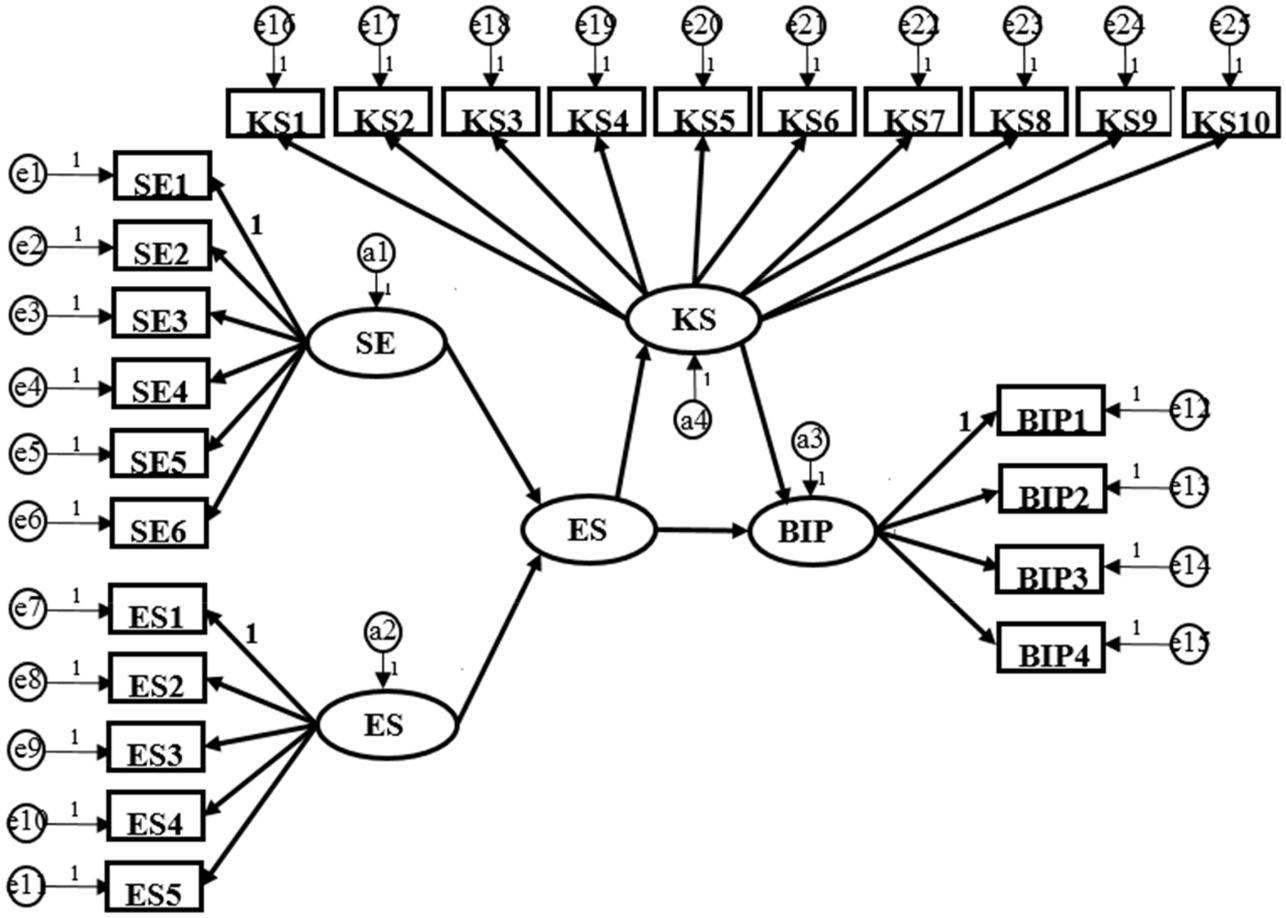
Analysis results of research model. e represents variance; a represents residual. SE, Self-Efficacy; ES, Emotional Stability; PC, Psychological Capital; KS, Knowledge Sharing; BIP, Breakthrough Innovation Performance.

This study will use AMOS26.0 software to analyze the structural equation model. In the structural equation model, using the sum or average of items in each dimension instead of each item as the indicator of the latent variable can reduce the number of parameters, improve the reliability of measurement indicators and enhance the stability of parameters. In this study, the above method will be used to calculate the average value of each latent variable and then analyze it. The final result is shown in Figure 2. The right part includes 4 items of breakthrough innovation performance and error items. The left part is two dimensions of psychological capital (self-efficacy and emotional stability) with 11 items and error items, and the upper part is 10 items and error items of intermediary knowledge sharing.

### 4.2. Hypothesis tests

#### Main effect test

That is to say, the relationship between independent variables and dependent variables is tested under the condition that the intermediate variables are not added into the model. The results show that employees’ self-efficacy can promote breakthrough innovation performance. Hypothesis 3 is verified (Table 5). Employee emotional stability can promote breakthrough innovation performance. Hypothesis 4 is verified (Table 6).

**Table 5:** Path regression coefficients of research model.

Variable relation	Estimate	SE	C.R.	*p*-Value
Breakthrough innovation performance ← Self-efficacy	0.833	0.79	10.491	***
Knowledge sharing ← Self-efficacy	0.881	0.57	15.383	***
Breakthrough innovation performance ← Emotional stability	0.862	0.74	10.826	***
Knowledge sharing ← Emotional stability	0.877	0.53	15.294	***
Breakthrough innovation performance ← Knowledge sharing	0.203	0.71	2.831	**

**p < 0.01, ***p < 0.001.

**Table 6:** Regression coefficient of main effect path.

Variable relation	Estimate	SE	C.R.	*p*-Value
Breakthrough innovation performance ← Self-efficacy	1.044	0.56	19.227	***
Breakthrough innovation performance ← Emotional stability	1.045	0.55	19.381	***

***p < 0.001.

#### Intermediary effect test

**Figure 3:**
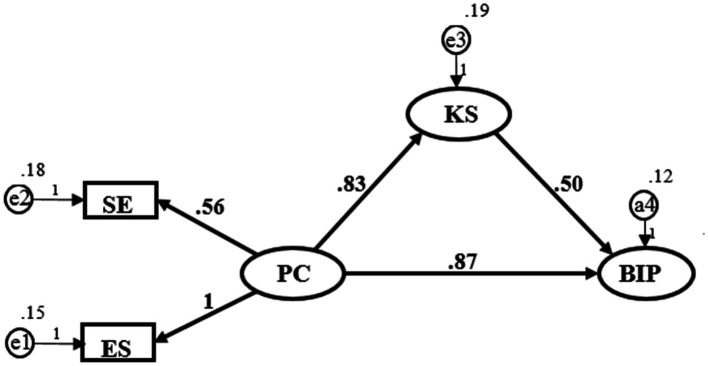
Research model path. SE, Self-Efficacy; ES, Emotional Stability; PC, Psychological Capital; KS, Knowledge Sharing; BIP, Breakthrough Innovation Performance.

The intermediate variables are added to the research model for overall model test (Figure 3). The regression coefficients of the whole model path are shown in Table 5. The results show that the two dimensions of psychological capital have significant positive impact on knowledge sharing (*β* = 0.881, *p* < 0.001; *β* = 0.877, *p* < 0.001), hypothesis 1 and hypothesis 2 are verified. Knowledge sharing has a significant positive impact on breakthrough innovation performance (*β* = 0.203, *p* < 0.01), hypothesis 5 is verified. In this model, the principal effect is still significant, but the coefficient has decreased, indicating that knowledge sharing acts as a partial mediator. Hypothesis 6 and 7 are verified

### 4.3. Testing intermediary effect with regression

Control variables were further added, and the variables were regressed by SPSS18.0 software. The regression results are shown in Tables 7, 8. Regression analysis was applied to test the relationship among the four variables (Tables 7, 8). In Table 7, Model 1 is a study on the impact of control variables on breakthrough innovation performance. The VIF (1.307. 3.111, 1.121, 4.325, 3.810) of the control variables are all <10, indicating that there is no serious collinearity problem in the model and the results are acceptable. The second model is the research on the influence of self-efficacy on the performance of breakthrough innovation, in which the *β* of self-efficacy is 0.717 (*p* < 0.001), indicating that employees’ self-efficacy can significantly promote the performance of breakthrough innovation. Model 3 introduces the variables of self-efficacy and knowledge sharing at the same time. The *β* value of self-efficacy is 0.566 (*p* < 0.001) and the *β* value of knowledge sharing is 0.363 (*p* < 0.001). both self-efficacy and knowledge sharing have significant impact on the performance of breakthrough innovation. At the same time, the *β* value of self-efficacy (0.556) is less than the *β* value before knowledge sharing (0.717), which shows that the influence of knowledge sharing on self-efficacy and breakthrough innovation performance is weakened, and knowledge sharing plays a part of intermediary role between self-efficacy and breakthrough innovation performance. These results again partially support H6.

**Table 7:** Regression analysis of self-efficacy and knowledge sharing on breakthrough innovation performance.

	Model one	Model two	Model three
Constant	12.279	1.029	0.558
Control variable			
Gender	−0.847	−0.413	−0.506
Marital status	−0.25	−0.083	−0.186
Culture	0.057	0.120	0.144
Working years	0.161	−0.003	−0.003
Age	−0.039	−0.098	−0.037
Independent variable			
Self-efficacy		0.717***	0.566***
Knowledge sharing			0.363***
*R* ^2^	0.038	0.704	0.731
Adjustment r^2^	0.018	0.697	0.724
*F*	1.885	99.901	101.200
Δ*R*^2^	0.038	0.666	0.027
Δ*F*	1.885	756.443	33.733

Dependent variable: Breakthrough Innovation Performance ***p < 0.001.

Model 1 is a study on the impact of control variables on breakthrough innovation performance; Model 2 is the research on the influence of self-efficacy on the performance of breakthrough innovation; Model 3 introduces the variables of self-efficacy and knowledge sharing at the same time.

**Table 8:** Regression analysis of emotional stability and knowledge sharing on breakthrough innovation performance.

	Model one	Model two	Model three
Constant	12.914	1.024	0.542
Control variable			
Gender	−0.832	−0.452	−0.514
Marital status	−0.25	−0.081	−0.142
Culture	0.052	0.121	0.157
Working years	0.198	−0.002	−0.001
Age	−0.031	−0.096	−0.044
Independent variable			
Emotional stability		0.726***	0.582***
Knowledge sharing			0.372***
*R* ^2^	0.038	0.704	0.713
Adjustment *R*^2^	0.011	0.615	0.741
*F*	1.825	99.915	102.210
Δ*R*^2^	0.031	0.431	0.026
Δ*F*	1.817	755.521	33.915

Dependent Variable: Breakthrough Innovation Performance ***p < 0.001.

Model 1 is a study on the impact of control variables on breakthrough innovation performance; Model 2 is the research on the influence of emotional stability on the performance of breakthrough innovation; Model 3 introduces the variables of emotional stability and knowledge sharing at the same time.

In Table 8, Model 1 is a study on the impact of control variables on breakthrough innovation performance. The VIF of the control variables (1.314. 3.173, 1.142, 4.317, 3.715) are all <10, indicating that there is no serious collinearity problem in the model and the results are acceptable. The second model is the research on the influence of emotional stability on the performance of breakthrough innovation, in which the *β* of emotional stability is 0.726 (*p* < 0.001), indicating that employees’ emotional stability can significantly promote the performance of breakthrough innovation. Model 3 introduces the variables of emotional stability and knowledge sharing at the same time. The *β* value of emotional stability is 0.582 (*p* < 0.001) and the *β* value of knowledge sharing is 0.372 (*p* < 0.001), indicating that both emotional stability and knowledge sharing have significant impact on the performance of breakthrough innovation. At the same time, the *β* value of emotional stability (0.582) is less than the *β* value before knowledge sharing (0.726), which shows that the influence of knowledge sharing on emotional stability and breakthrough innovation performance is weakened, and knowledge sharing plays a part of intermediary role between emotional stability and breakthrough innovation performance. These results again partially support H7.

## 5. Discussion

### 5.1. Conclusion

Firstly, employees’ psychological capital has a significant positive impact on breakthrough innovation performance. And psychological capital is a positive state, including self-efficacy and emotional stability. Self-efficacy is to believe in oneself and affirm one’s own ability, and emotional stability is related to individual recovery and growth. It refers to effective response and adaptation in the face of loss, difficulty or adversity. At the same time, it emphasizes individual growth after setbacks. The employees are engaged in deep-level work, and the respondents of this questionnaire are all employees engaged in IT, testing, R&D, management and other positions. If they do not have a positive mental state, they will not be able to better face challenges and setbacks at work, nor will they be able to promote breakthrough innovation performance. The positive mentality and psychological ability of employees can promote the formation of breakthrough innovation ability and the realization of breakthrough innovation idea.

Secondly, employees’ psychological capital has a significant positive impact on knowledge sharing behavior. Employees with higher psychological capital are more willing to communicate and transfer information with colleagues, which is beneficial to knowledge sharing. Setbacks are inevitable in the process of knowledge sharing. Optimistic employees can smile and face setbacks. Hopeful employees are willing to participate in the knowledge sharing process to achieve their goals and employees with self-confidence are full of confidence in their work goals. They believe that they have the ability to face setbacks in the process of achieving the goals and boldly express their thoughts. Employees with good emotional stability can correctly handle the difficulties in the information transmission process, and actively adjust themselves after the setbacks, and quickly return to work. Employees with strong psychological capital are more likely to get along well with others. They dare to communicate, so that they can gain more knowledge and experience and improve their ability.

Thirdly, employee’s knowledge sharing behavior has significant positive impact on breakthrough innovation performance. Knowledge sharing not only emphasizes the sharing of knowledge and skills with members within the organization, but also encourages employees to communicate and learn with members outside the organization. With the arrival of big data era, data processing begins to be completed through cloud computing. We need to find the information we need from big data and turn it into knowledge. The foundation of breakthrough innovation is knowledge, and the subject of breakthrough innovation is employees. Knowledge sharing becomes an indispensable condition in the process of breakthrough innovation. Breakthrough innovation mainly comes from the team. Without the communication and learning of team members, it is difficult to achieve breakthrough innovation.

Fourthly, knowledge sharing plays an intermediary role in psychological capital and breakthrough innovation performance. Psychological capital can not only directly affect employees’ breakthrough innovation performance, but also indirectly affect breakthrough innovation performance through knowledge sharing. Psychological capital is a kind of positive mentality of employees. It is not enough to have a positive mentality. It should be reflected in actions in the end. Only “I can do it” can “I really do it.” The process of knowledge sharing is not simple. We need the recognition and trust of others in order to give full play to the power of knowledge sharing.

Comparison with other studies (Larson and Luthans, 2008; Carr, 2008; Qingsong and Daming, 2010; Yuan and Jun, 2016; Qing et al., 2022), reflection on the results are as follows: this paper studies the relationship among psychological capital, knowledge sharing and breakthrough innovation performance, but the impact of breakthrough innovation performance is complex and diverse in reality. For example, personal perspective (employee well-being, job satisfaction, emotional intelligence, personal expectations and personality, etc.) and situational perspective (organizational incentive mechanism, organizational atmosphere, organizational leadership style, organizational learning ability and corporate culture, etc.) will affect the performance of breakthrough innovation. In addition, the related factors that affect psychological capital and knowledge sharing, as well as the interaction between the various influencing factors are not considered. The follow-up research needs to further enrich the research model and consider more variables that affect employees’ psychological capital, knowledge sharing and breakthrough innovation performance and the interaction between these variables. In addition, due to different research objects, the dimensions of relevant variables are different and need to be further enriched and improved.

### 5.2. Theoretical contributions

Firstly, in terms of factors affecting employees’ breakthrough innovation performance, more attentions are paid to individuals (employee well-being, emotional intelligence and work involvement, etc.) and organizational aspects (organizational atmosphere, learning ability, corporate tasks, etc.) in the past. This study focuses on the middle-level and senior-level management of high-tech enterprises and grass-roots employees who are committed to research and development. It also focuses on employees’ psychological capital and knowledge sharing. In addition, it explores its impact on breakthrough innovation performance from both psychological and action levels. It enriches the research field of innovation management.

Secondly, this study constructs and verifies the model of psychological capital, employee breakthrough innovation performance and the relationship between them, and fully explores employees’ breakthrough innovation potential from the psychological aspect of employees in order to improve the breakthrough innovation performance of the whole company. At the same time, this study also introduces knowledge sharing, which is beneficial to high-tech enterprises to create a strong learning atmosphere and lay a solid foundation for employee’s breakthrough innovation. In addition, it also pays attention to the psychological level and the action level of employees, which provides management enlightenment for high-tech enterprises to cultivate breakthrough innovation of employees. It should be pointed out that 1/3 of the survey objects in this study are grassroots employees committed to research and development. These samples cannot obtain enough information of the firms level, thus this study also has certain limitations.

### 5.3. Managerial implications

Firstly, consider paying attention to the improvement of employees’ psychological capital. On the one hand, improve self-efficacy. For example, it can improve employees’ ability to express their opinions about the company’s plan with confidence, to get rid of work difficulties, to analyze and solve problems with confidence, and so on. On the other hand, keep emotional stability. This requires identifying one’s own position and setting an expected goal at each stage. Only after reaching the goal can we enjoy the pleasure of life and remain optimistic.

Secondly, consider improve the knowledge sharing conditions of employees. On the one hand, improve the “hardware” factors of knowledge sharing. “Hardware” can be divided into two categories, one is the necessary facilities to enhance employees’ knowledge reserve, the other is a communication tool to enrich employee communication. The premise of knowledge sharing is to have “a certain knowledge reserve.” Set courses they are interested in according to employees’ wishes, so as to increase the “class attendance rate” of employees. The improvement of communication tools and technology can also improve the quality of knowledge sharing among employees. For example, communication tools such as employees’ internal email, internal telephone, internal QQ and internal forum can overcome the obstacles of time and space, receive information from employees at any time, and provide conditions for employees to “speak out.” However, it is not enough to only receive internal information. Employees also need to participate in external communication. Information technology enables us to better communicate with the outside world. More and more employees with the same interests, such as “video conference,” “online studio” and “online classroom,” are connected and communicate with each other. On the other hand, improve the “software” factor of knowledge sharing to motivate employees to share knowledge. Companies need to select employee with strong knowledge acquisition ability and carry out a series of activities to cultivate employee’s awareness of knowledge sharing. Establishing a learning organization is a way to speed up the flow of knowledge.

### 5.4. Limitations and suggestions for future research

Firstly, the sample is taken from the employees engaged in IT, testing and certification, R&D, management and other positions from Beijing-Tianjin-Hebei region. Therefore, the sample coverage has certain limitations. In addition, the influence of the size and nature of the company is not considered. The follow-up research needs to enrich the source and quantity of samples and consider more regions, positions and companies to make the research more universal.

Secondly, this paper studies the relationship among psychological capital, knowledge sharing and breakthrough innovation performance, but the impact of breakthrough innovation performance is complex and diverse in reality. For example, personal perspective (employee well-being, job satisfaction, emotional intelligence, personal expectations and personality, etc.) and situational perspective (organizational incentive mechanism, organizational atmosphere, organizational leadership style, organizational learning ability and corporate culture, etc.) will affect the performance of breakthrough innovation. In addition, the related factors that affect psychological capital and knowledge sharing, as well as the interaction between the various influencing factors are not considered. The follow-up research needs to further enrich the research model and consider more variables that affect employees’ psychological capital, knowledge sharing and breakthrough innovation performance and the interaction between these variables. In addition, due to different research objects, the dimensions of relevant variables are different and need to be further enriched and improved.

## Data availability statement

The original contributions presented in the study are included in the article/supplementary material, further inquiries can be directed to the corresponding author.

## Ethics statement

Ethical review and approval was not required for the study on human participants in accordance with the local legislation and institutional requirements. Written informed consent from the participants was not required to participate in this study in accordance with the national legislation and the institutional requirements.

## Author contributions

YL contributed the central idea, analyzed most of the data, and wrote the initial draft of the paper. JC contributed to refining the ideas, carrying out additional analyses, and finalizing this paper. All authors contributed to the article and approved the submitted version.

## Funding

This study was supported by Beijing Municipal Natural Science Foundation Project of China (grant no. 9222012). The authors thank it wholeheartedly for funding our research.

## Conflict of interest

XH was employed by CRRC Industrial Academy Co., Ltd.

The remaining authors declare that the research was conducted in the absence of any commercial or financial relationships that could be construed as a potential conflict of interest.

## Publisher’s note

All claims expressed in this article are solely those of the authors and do not necessarily represent those of their affiliated organizations, or those of the publisher, the editors and the reviewers. Any product that may be evaluated in this article, or claim that may be made by its manufacturer, is not guaranteed or endorsed by the publisher.
